# Human NK Cells Develop an Exhaustion Phenotype During Polar Degranulation at the *Aspergillus fumigatus* Hyphal Synapse

**DOI:** 10.3389/fimmu.2018.02344

**Published:** 2018-10-22

**Authors:** Virginia Santiago, Katayoun Rezvani, Takuya Sekine, Justin Stebbing, Peter Kelleher, Darius Armstrong-James

**Affiliations:** ^1^Faculty of Medicine, Imperial College London, National Heart and Lung Institute, London, United Kingdom; ^2^Stem Cell Transplantation and Cellular Therapy, MD Anderson Cancer Centre, Houston, TX, United States; ^3^Kennedy Institute, University of Oxford, Oxford, United Kingdom; ^4^Department of Surgery and Cancer, Faculty of Medicine, Imperial College London, London, United Kingdom; ^5^Faculty of Medicine, Centre for Immunology and Vaccinology, Imperial College London, London, United Kingdom

**Keywords:** NK cells, *Aspergillus fumigatus*, fungal disease, immunocompromise, leukaemia

## Abstract

Pulmonary aspergillosis is an opportunistic fungal infection affecting immunocompromised individuals. Increasing understanding of natural killer (NK) cell immunobiology has aroused considerable interest around the role of NK cells in pulmonary aspergillosis in the immunocompromised host. Murine studies indicate that NK cells play a critical role in pulmonary clearance of *A. fumigatus*. We show that the *in vitro* interaction between NK cells and *A. fumigatus* induces partial activation of NK cell immune response, characterised by low-level production of IFN-γ, TNF-α, MIP-1α, MIP-1β, and RANTES, polarisation of lytic granules and release of fungal DNA. We observed a contact-dependent down-regulation of activatory receptors NKG2D and NKp46 on the NK cell surface, and a failure of full granule release. Furthermore, the NK cell cytokine-mediated response to leukaemic cells was impaired in the presence of *A. fumigatus*. These observations suggest that *A. fumigatus*-mediated NK cell immunoparesis may represent an important mechanism of immune evasion during pulmonary aspergillosis.

## Introduction

Pulmonary aspergillosis is a high mortality fungal infection in immunocompromised hosts. Whilst significant progress has been made with antifungal therapies, drug resistance is emerging (1) and there is still a pressing need for novel immunotherapies to improve outcomes (2). Neutrophils play a primary role in host immunity during *Aspergillus fumigatus* germination in the lung, however studies in murine models show that recruitment of NK cells to the lungs is critical for early host defence (3, 4) and that NK cells are the major source of IFN-γ in the lungs during murine neutropaenic aspergillosis (4).

Clinical studies have also shown that exogenous IFN-γ is beneficial as adjunctive therapy in pulmonary aspergillosis (5, 6). Initial studies suggested that unstimulated or IL-2 stimulated human NK cells kill *A. fumigatus* hyphae but not conidia, through perforin-dependent cytotoxicity. This was associated with reduced levels of IFN-γ and GM-CSF production (7). However, a further study found that IL-2 pre-stimulated human NK cells release IFN-γ and TNF-α in response to *A. fumigatus* germlings. NK cell—mediated damage of *A. fumigatus* germlings was independent of NK cell degranulation and instead found to be a consequence of direct IFN-γ-mediated fungal damage(8).

We sought to systematically define human NK cell interactions with *A. fumigatus*, and the key NK cell responses that ensue. We found that co-culture of NK cells with *A. fumigatus in vitro* results in tight adhesion of the cells to the fungus, F-actin accumulation at the immune synapse and granule polarisation. This was associated with reduced surface expression of CD56 and the activating receptors NKG2D and NKp46. We confirmed that *A. fumigatus* co-culture did lead to low-level NK cell IFN-γ or TNF-α release, as well as significant production of MIP-1α, MIP-1β, and RANTES. Furthermore, in the presence of *A. fumigatus*, NK cell cytokine responses to leukaemic cells were impaired. NK cells did not reduce fungal viability or inhibit fungal metabolism, but were able to induce fungal DNA release which may represent an important mechanism for activation of subsequent systemic immune responses through nucleic-acid-sensing pathways. These studies show that NK cell adhesion to *A. fumigatus* results in a exhaustion phenotype associated with activatory receptor downregulation, impaired degranulation and cytokine responses, and impaired antifungal cytotoxicity.

## Materials and methods

### Fungal strains and culture

*A. fumigatus* ATCC46645 WT (American Type Culture Collection, Manassas, VA) was used for ELISA, Luminex Multiplex (Luminex, Austin, TX) and FACS experiments. *A. fumigatus* ATCC 46645-GFP was a kind gift from Professor Frank Ebel and it was used for confocal microscopy experiments. Germlings were generated from resting conidia in RPMI at 1 × 10^6^ conidia/mL at 37°C for 6 h. Germlings and hyphae were fixed in 2% formalin overnight at 4°C, quenched in 0.1 M ammonium chloride for 10 min and 4 washes in PBS. To prepare fungal culture supernatants, *A. fumigatus* ATCC46645 was incubated for 16 h at 5 × 10^5^ conidia/mL. Resting conidia were 2–3 μm in size, swollen conidia were 5–10 μm in size, and round, and germlings were defined by the appearance of a small protuberance on the conidial cell wall. Hyphae were defined as multicellular and multinucleated structures, internally divided in compartments separated by internal cross-walls called septa.

### Isolation of human natural killer cells

NK cells were isolated from peripheral blood mononuclear cells from healthy volunteers or from leukodepletion samples from AML patients (following informed consent and under IRB approved protocols, MD Anderson Cancer Center) by Ficoll-Paque gradient centrifugation and negative magnetic bead isolation (NK cell isolation kit human; Miltenyi Biotec, Auburs, CA). NK cells were cultured in DMEM containing 10% human serum (Life Technologies), 1% sodium pyruvate (Sigma-Aldrich), 1% non-essential amino acids (Gibco by Life Technologies), 50 μM mercaptoethanol (Gibco by Life Technologies), and either 300 U/mL (standard dose) or 1000 U/mL (high dose) of recombinant human IL-2 (Peprotech), unless otherwise specified.

### ELISA and luminex assays

NK cells were plated at 2 × 10^5^ cells per well in a 96-well flat bottom plate and stimulated with live *A. fumigatus* germlings at specified MOIs. Analytes were measured in supernatants using the DuoSet human ELISA kits (R and D Systems). Luminex analysis of supernatants was performed using the Milliplex human cytokine/chemokine magnetic bead panel kit (Merck Millipore).

### Flow cytometry analysis of NK cell interaction with *A. fumigatus*

NK cells (2 × 10^5^ cells/well) were cultured in 96-well flat bottom plates and challenged with *A. fumigatus*. After co-incubation, cells were filtered using 100 μm cell strainer to remove hyphae. Cell surface staining was performed by incubation with relevant antibodies for 25 min in the dark. Cells were washed in PBS, followed by staining with Zombie-aqua cell viability stain (BioLegend, UK) according to the manufacturer's instructions. A washing step was performed followed by cell permeabilisaton and fixation using permeabilising solution 2 (BD Biosciences). To determine intracellular expression of IFN-γ and TNF-α, 5 μg/mL of brefeldin A was added during co-incubation. For intracellular staining, cells were incubated with IFN-γ and TNF-α antibodies after the permeabilisation and fixation step. For degranulation assays, CD107a antibody was added to the co-cultures at time 0. The following antibodies were used: CD56 (PE Cy7); CD3 (APC H7); CD16 (Alexa Flour 647); NKG2D (PE); NKp46 (PerCP Cy5.5);NKp80 (PE); CD107a (FITC); IFN-γ (V450); TNF-α (PerCP-Cy5.5) (all Biolegend). For analysis of CD56 internalisation, NK cells were incubated overnight with *A. fumigatus*. After the co-incubation period, cells were collected, washed with PBS and surface staining performed for 25 min at room temperature in the dark. Cells were then washed and stained with Zombie-aqua cell viability dye. A washing step was performed followed by permeabilisation and fixation using permeabilising solution 2 (BD Biosciences). After permeabilisation step, cells were washed and incubated once more with CD56 antibody for an extra 25 min. Cells were acquired on Fortessa analyser (BD Biosciences) and data was analysed with FlowJo Software.

### Confocal microscopy

GFP-*A. fumigatus* germlings were propogated on coverslips, NK cells added at an MOI of 0.05 and co-incubated overnight, then washed twice with PBS and fixed and permeabilised in permeabilising solution 2 (BD Biosciences). Cells were washed with PBS, blocked in PBS containing 10% goat serum (2 h, room temperature) and incubated overnight at 4°C with a primary antibody (anti-LAMP-1, clone H4A3, Biolegend; anti-perforin, clone dG9, Biolegend; anti-granulysin, Santa Cruz) in blocking buffer. After washing with PBS, cells were incubated with anti-mouse Alexa Fluor® 555 (Biolegend) and Alexa Fluor® 633 phalloidin (Life Technologies) diluted 1:500 for 1 h at room temperature in the dark. Cells were washed with PBS and mounted with Vectashield mounting medium containing DAPI (Vector laboratories). Images were captured on a Zeiss LSM-510 confocal microscope.

### Analysis of granule polarisation

Quantitative analysis of granule polarisation toward *A. fumigatus* was calculated using a Matlab code developed by Dr. Yuriy Alexandrov (FungusDependentGranuleRelease_tools). This calculates the cosine of the angle between the direction of a line from the granule to the fungus and the direction of a line from the granule to the cell centre. Granules located in the cell on the opposite side of the fungus have cosine close to 1, whereas granules polarised to the fungus have cosine close to −1.

### Analysis of NK cell-mediated fungal damage

Human NK cells were incubated with *A. fumigatus* at a MOI 0.05 overnight in μ-slide 8 well plates (Ibidi) at a density of 3 × 10^5^ NK cells to 1.5 × 10^4^ *A. fumigatus* germlings per well. Propidium iodide was added to each well (1:500) and images captured by confocal microscopy. Fungal gDNA release was quantified in co-culture supernatant by quantitative real-time PCR. NK cells were incubated with *A. fumigatus* germlings at a MOI 0.05. Co-culture was performed overnight in a 24 well plate, incubating 1 × 10^6^ NK cells with 5 × 10^4^ *A. fumigatus* germlings per well. Then supernatant was collected and centrifuged at 2000 rpm for 5 min to pellet possible cells and fungi in suspension, and stored at −80°C. Quantification was performed as described (9) with an *Aspergillus*-specific probe FAM-CATTCGTGCCGGTGTACTTCCCCG-TAMRA (Sigma-Aldrich).

### XTT assay

XTT (2,3-bis(2-methoxy-4-nitro-5-[(sulfophenylamino)carbonyl]-2H-tetrazolium-hydroxide) assay for assessment of fungal cell damage was performed as described previously (10). *A. fumigatus* were seeded in a 96 well plate at a density of 1 × 10^4^ conidia per well until they form germlings. NK cells were added at an MOI 0.05 and the co-incubation was performed overnight. Amphotericin B was used as positive control for fungal killing at a concentration of 1 μg/mL. After the incubation period, fungal damage was assessed using the XTT assay.

### Quantification of galactomannan

Quantification of galactomannan in the supernatant of co-cultures, was performed using the PLATELIA™ *Aspergillus* galactomannan enzyme immunoassay (Bio-Rad) according to the manufacturers instructions.

### Statistical analysis

Data were presented as mean ± SEM and were analysed using GraphPad Prism software (version 6.0; GraphPad). Significance was determined using a paired or unpaired Student's *t*-test; ^*^P ≤ 0.05; ^**^P ≤ 0.01; ^***^P ≤ 0.001.

## Results

### Primary human NK cells produce low levels of cytokines and chemokines during challenge with *A. fumigatus*

**Figure 1:**
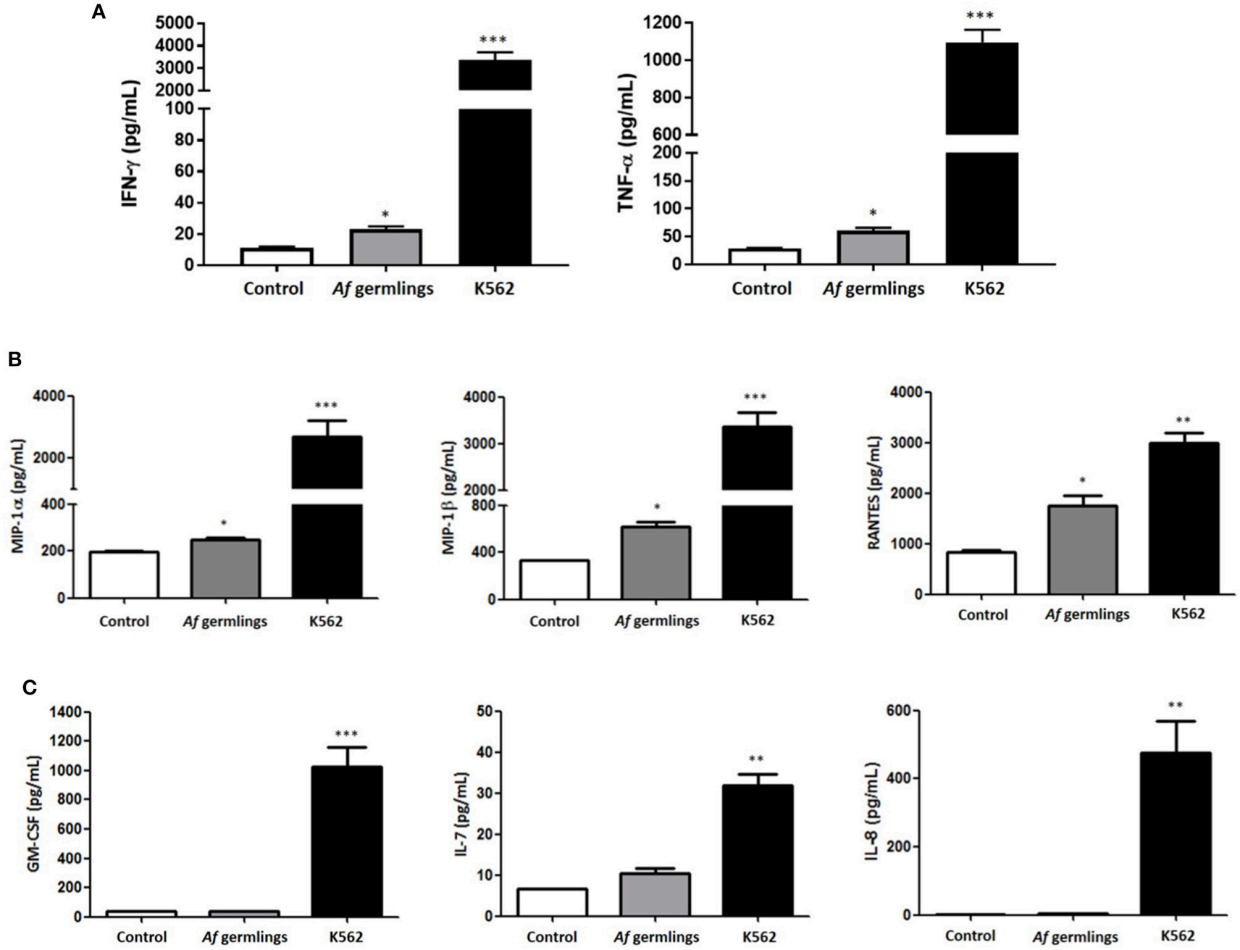
Primary human NK cells secrete IFN-γ, TNF-α, MIP-1α, MIP-1β, and RANTES in response to *A. fumigatus* germlings **(A)**. Analysis of cytokine production by NK cells in response to *A. fumigatus*. Primary human NK cells were incubated overnight with *A. fumigatus* germlings at MOI = 1 in the presence of IL-2. Co-incubation with K562 cell line at MOI 1 was used as positive control. IFN-γ and TNF-α levels were determined in the supernatant of co-cultures by Luminex multiplex assay. Bar graphs show the mean + SEM of IFN-γ and TNF-α production. Statistical analysis was performed using the Student's unpaired *t*-test. *p < 0.05, ***p < 0.001. **(B)** NK cells produce MIP-1α, MIP-1β, and RANTES in response to *A. fumigatus* germlings in the presence of IL-2. Primary human NK cells were incubated overnight with *A. fumigatus* germlings at MOI = 1 in the presence of IL-2. Co-incubation with K562 at MOI = 1 was used as positive control. MIP-1α, MIP-1β, and RANTES levels were determined in the supernatant of co-cultures by Luminex multiplex assay. Bar graphs show the mean + SEM of MIP-1α, MIP-1β, and RANTES production. Statistical analysis was performed using the Student's unpaired *t*-test. *p < 0.05, ** *p* < 0.01, *** *p* < 0.001. *n* = 3 **(C)**. Analysis of GM-CSF, IL-7, and IL-8 production in response to *A. fumigatus*. Primary human NK cells were incubated overnight with *A. fumigatus* germlings at MOI = 1 in the presence of IL-2. Co-incubation with K562 at MOI = 1 was used as positive control. GM-CSF, IL-7, and IL-8 levels were determined in the supernatant of co-cultures by Luminex multiplex assay. Bar graphs show the mean + SEM of GM-CSF, IL-7, and IL-8 production. Statistical analysis was performed using the Student's unpaired *t*-test. *p < 0.05, **p < 0.01, ***p < 0.001. *n* = 3.

NK cells were incubated with *A. fumigatus* germlings and cytokine and chemokine production quantified in supernatants by Luminex multiplex assay. In the presence of IL-2 there was a very small but significant increase in IFN-γ and TNF-α (Figure 1A). There was a small but significant increase in MIP-1α, MIP-1β, and RANTES levels after 12 h incubation (Figure 1B). There was no significant changes in GM-CSF, IL-7, or IL-8 (Figure 1C). In presence of IL-2 and IL-15 there was a significant increase in MIP-1α levels and RANTES had near significance (*P* = 0.0512) at 12 h post-infection (p.i.) (data not shown).

### NK cell adhesion to *A. fumigatus* leads to F-actin accumulation and granule polarisation at the immune synapse

**Figure 2:**
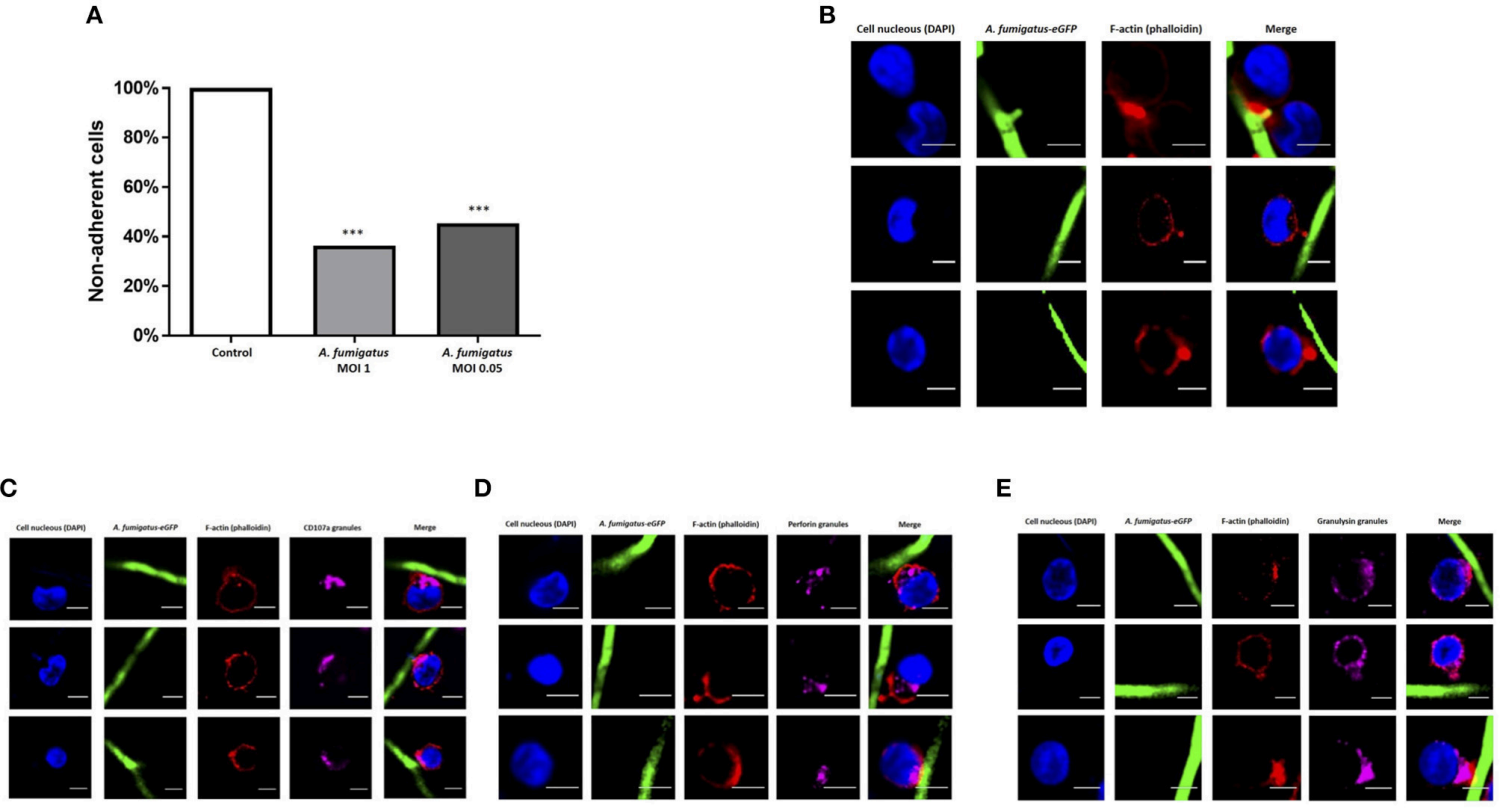
NK cell adhesion to *A. fumigatus* leads to F-actin accumulation at the immune synapse and granule polarisation **(A)**. NK cells strongly adhere to *A. fumigatus* when co-incubated with the fungus *in vitro*. Primary human NK cells were incubated with *A. fumigatus* overnight. After the incubation period cells were collected and filtered through a 100 μm cell strainer to exclude the hyphae and all NK cells attached to the fungus. The number of cells was determined by FACS. Bar graph shows the mean + SEM. This data is representative of 10 independent experiments. Statistical analysis was performed using the unpaired Student's *t*-test. ***P < 0.001 **(B)**. Filamentous actin accumulates at the immune synapse formed between NK cells and *A. fumigatus*. Representative images of primary human NK cells incubated with *A. fumigatus* GFP germlings at MOI = 0.05. Co-culture was performed overnight, cells were then washed, fixed, permeabilised, and incubated in blocking buffer. F-actin staining was performed incubating the cells with Alexa Fluor 633 phalloidin (Dil 1:500) for 1 h at room temperature. Cover slips were washed 3 times in PBS before being mounted with Vectashield mounting medium containing DAPI. Phalloidin staining is indicated in red, the nuclei are indicated in blue and *A. fumigatus* GFP is shown in green. Scale bars = 5 μm. **(C)** NK cell granules polarise toward *A. fumigatus in vitro*. Representative images of primary human NK cells incubated overnight with *A. fumigatus* GFP germlings at MOI = 0.05. After the incubation period, cells were washed, fixed, permeabilised and incubated overnight with anti-human CD107a antibody at 4°C. Cells were then washed in PBS, followed by the incubation with Alexa Fluor 555 goat anti-mouse secondary antibody and Alexa Fluor 633 phalloidin for 1 h at room temperature. Cover slips were washed 3 times in PBS before being mounted with Vectashield mounting medium containing DAPI. Phalloidin staining is indicated in red, the nuclei are indicated in blue, CD107a staining is indicated in magenta and *A. fumigatus* GFP is shown in green. Scale bars = 5 μm. **(D)** NK cell perforin granules polarise toward *A. fumigatus in vitro*. Representative images of primary human NK cells incubated overnight with *A. fumigatus* GFP germlings at MOI = 0.05. After the incubation period, cells were washed, fixed, permeabilised, and incubated overnight with anti-human perforin antibody at 4°C. Cells were then washed in PBS, followed by the incubation with Alexa Fluor 555 goat anti-mouse secondary antibody and Alexa Fluor 633 phalloidin for 1 h at room temperature. Cover slips were washed 3 times in PBS before being mounted with Vectashield mounting medium containing DAPI. Phalloidin staining is indicated in red, the nuclei are indicated in blue, perforin staining is indicated in magenta and *A. fumigatus* GFP is shown in green. Scale bars = 5 μm. **(E)** NK cell granulysin granules polarise toward *A. fumigatus in vitro*. Representative images of primary human NK cells incubated overnight with *A. fumigatus* GFP germlings at MOI = 0.05. After the incubation period, cells were washed, fixed, permeabilised and incubated overnight with anti-human granulysin antibody at 4°C. Cells were then washed in PBS, followed by the incubation with Alexa Fluor 555 goat anti-mouse secondary antibody and Alexa Fluor 633 phalloidin for 1 h at room temperature. Cover slips were washed 3 times in PBS before being mounted with Vectashield mounting medium containing DAPI. Phalloidin staining is indicated in red, the nuclei are indicated in blue, granulysin staining is indicated in magenta and *A. fumigatus* GFP is shown in green. Scale bars = 5 μm.

During co-culture experiments we microscopically observed tight adhesion of NK cells to *A. fumigatus* hyphae. To further quantify this, NK cells were co-incubated with *A. fumigatus*, cells were collected at specified time points, filtered to exclude the hyphae and adhered NK cells, and quantified by multiparamete flow cytometry. This revealed that over 50% of the cells were adherent to the fungus (Figure 2A). As F-actin re-organisation is one of the first steps of immune synapse formation (11), we assessed F-actin accumulation during NK cell adherence to *A. fumigatus* hyphae. F-actin was clearly observed to accumulate at the interface formed between NK cells and *A. fumigatus* (Figure 2B).

To assess whether formation of synapses led to F-actin-associated polarisation of lytic granules, NK cells were incubated overnight with germlings at an MOI of 0.05, followed by intracellular staining of CD107a, perforin and granulysin. Granule polarisation was observed at synapses (Figures 2C–E).

**Figure 3:**
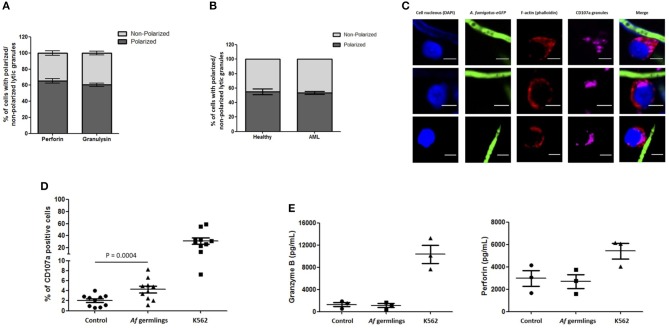
NK cell granule release is impaired during direct contact with *A. fumigatus* hyphae **(A)**. Percentage of perforin- and granulysin-containing granules polarisation in NK cells co-incubated with *A. fumigatus*. The graph displays the percentage of perforin and granulysin polarisation toward *A. fumigatus*. Isolated primary human NK cells were challenged with *A. fumigatus* overnight at MOI = 0.05. Cells were then fixed, stained for perforin or granulysin and analysed by confocal microscopy. Bar graph shows the mean ± SD of three independent experiments. **(B)** AML NK cell incubation with *A. fumigatus* induces normal granule polarisation toward the fungus. NK cells isolated from healthy donors or from AML patients were challenged with *A. fumigatus* overnight at MOI = 0.05. After the incubation period, cells were fixed, stained for CD107a and analysed by confocal microscopy. The graph displays the percentage of CD107a polarisation toward *A. fumigatus*. Bar graph shows the mean ± SD of three independent experiments. **(C)** AML NK cell granules polarise toward *A. fumigatus in vitro*. Representative images of primary human NK cells from AML patients incubated overnight with *A. fumigatus* GFP germlings at MOI = 0.05. After the incubation period, cells were washed, fixed, permeabilised, and stained for CD107a. Phalloidin staining is indicated in red, the nuclei are indicated in blue, CD107a staining is indicated in magenta and *A. fumigatus* GFP is shown in green. Scale bars = 5 μm. **(D)** NK cell challenge with *A. fumigatus* leads to increased cell surface CD107a expression. Isolated human NK cells were incubated overnight with *A. fumigatus* germlings at MOI = 0.05. Co-incubation with K562 cell line at MOI = 1 was used as positive control for NK cell degranulation. CD107a antibody was added at time 0 h. After the incubation time, the cells were collected and washed with PBS. Cells were stained with surface antibodies, followed by the staining with Aqua zombie dye. CD107a expression by NK cells was determined by FACS analysis. Graphs show the mean ± SEM of CD107a expression. Statistical analysis was performed using the Student's paired *t*-test. *n* = 10 **(E)** NK cells do not secrete granzyme B and perforin in co-culture with *A. fumigatus*. Isolated human NK cells were incubated overnight with *A. fumigatus* germlings at MOI = 0.05. Co-incubation with K562 cell line at MOI = 1 was used as positive control for NK cell degranulation. Granzyme B and perforin levels were measured in the culture supernatant by ELISA. Graphs show the mean ± SEM of granzyme B and perforin production. Statistical analysis was performed using the Student's paired *t*-test. *n* = 3.

An average of 65.4% of NK cells expressing perforin and 60.5% of NK cells expressing granulysin had granules polarised toward *A. fumigatus* (Figure 3A). These changes were not impaired in AML patient NK cells (Figures 3B,C). FACS analysis showed a significant increase in degranulation when NK cells were incubated with *A. fumigatus* (Figure 3D). However there were no increases in perforin and granzyme B in the supernatant of co-cultures (Figure 3E).

### NK cell challenge with *A. fumigatus* results in a contact-dependent CD56 internalisation

**Figure 4:**
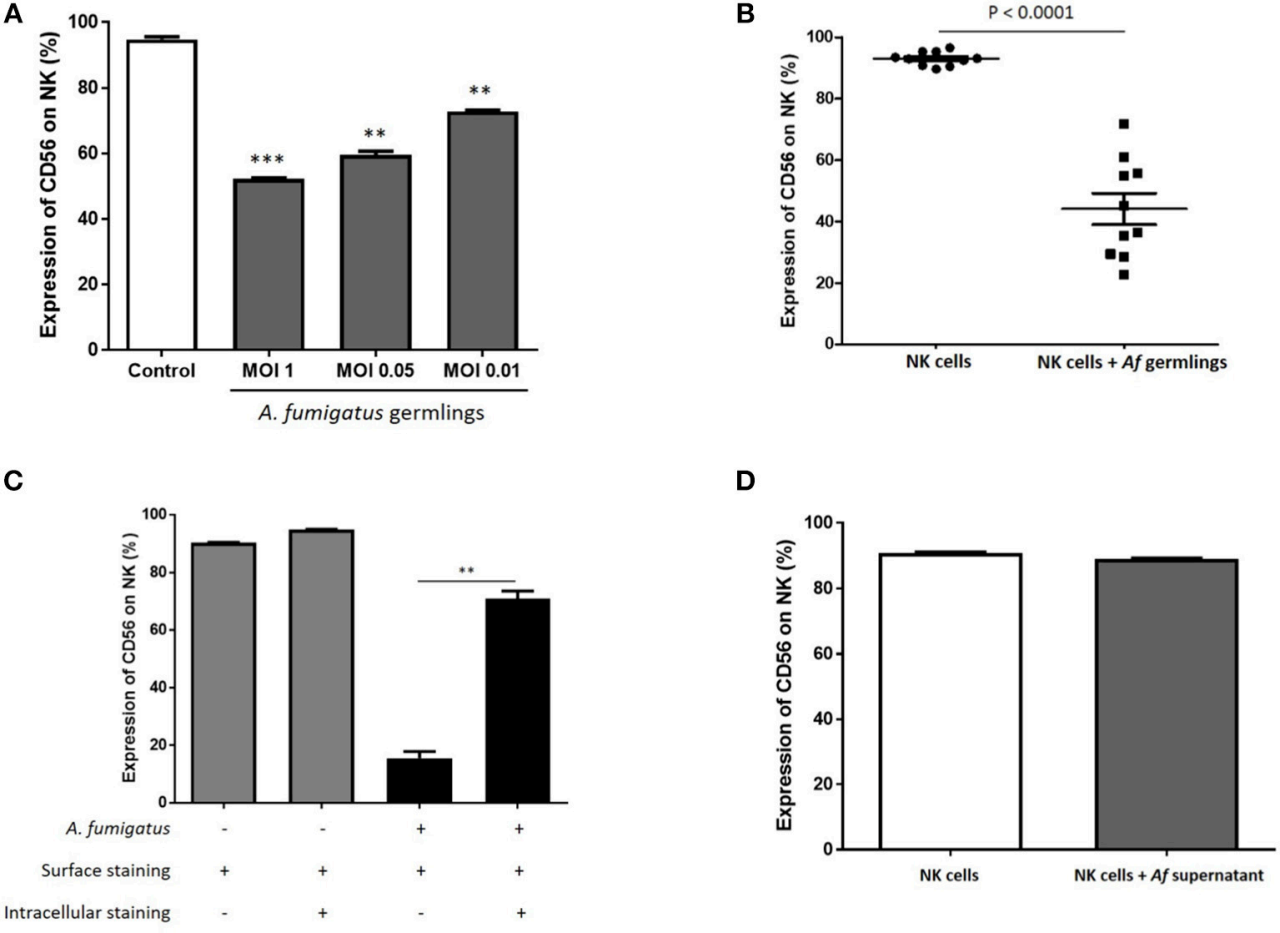
NK cell challenge with live *A. fumigatus* results in a contact-dependent reduction in CD56 surface expression as a result of CD56 internalisation **(A)**. CD56 NK cell surface expression is reduced in the presence of *A. fumigatus*. Human NK cells were incubated overnight with *A. fumigatus* germlings at MOI = 1, MOI = 0.05, and MOI = 0.01. CD56 expression was determined by FACS, gating live NK cells. Bar graph shows the mean + SEM of CD56 expression on NK cells. Statistical analysis was performed using the Student's unpaired *t*-test. **p < 0.01, ***p < 0.001. This data is representative of three independent experiments. **(B)** *A. fumigatus* co-incubation consistently reduces NK cells surface CD56 expression. Human NK cells from 10 different healthy donors were incubated overnight with *A. fumigatus* germlings at MOI = 0.05. CD56 expression was determined by FACS, gating live NK cells. Statistical analysis was performed using the Student's paired *t*-test. *n* = 10 ***p < 0.001 **(C)**. *A. fumigatus* induces CD56 internalisation on NK cells. Human NK cells were incubated overnight with *A. fumigatus* germlings at MOI = 0.05. NK cells were stained extracellularly or extracellularly and intracellularly with CD56 antibody. CD56 expression was determined by FACS, gating live NK cells. Bar graph shows the mean + SEM of CD56 expression on NK cells. This data is representative of three independent experiments. Statistical analysis was performed using the Student's unpaired *t*-test. **p < 0.01 **(D)**. CD56 downregulation on NK cell surface is dependent on direct contact with *A. fumigatus*. Human NK cells were incubated overnight with *A. fumigatus* culture supernatant. CD56 expression was determined by FACS, gating live NK cells. Bar graph shows the mean + SEM of CD56 expression on NK cells. This data is representative of three independent experiments. Statistical analysis was performed using the Student's unpaired *t*-test.

CD56 antigen is thought to be involved in NK-target cell interactions and killing (12, 13). Co-incubation of NK cells with *A. fumigatus* germlings led to a reduction in NK cells expressing CD56 (Figure 4A). Downregulation of CD56 on NK cells incubated with *A. fumigatus* was observed on both CD56^bright^ CD16^−^ and CD56^dim^ CD16^+^ NK cell subpopulations (Figures S1, S2). This was confirmed in 10 healthy donors (Figure 4B). This suggested that CD56 antigen was either internalised or degraded. To quantify CD56 internalisation, intra-cellular and extra-cellular staining was performed. This revealed that around 60% of CD56 antigen is internalised during *A. fumigatus* co-incubation (Figure 4C). To determine if CD56 downregulation was contact-dependent, NK cells were incubated with *A. fumigatus* culture supernatant. This did not result in CD56 downregulation on NK cell surface (Figure 4D), suggesting that CD56 downregulation is dependent on direct contact between NK cells and the fungus. Further studies revealed that this phenomenon is not induced by fixed *A. fumigatus* (data not shown) indicating that CD56 internalisation in NK cells is dependent on pathogen viability.

### NK cell challenge with *A. fumigatus* reduces surface expression of the activating receptors NKG2D and NKP46

**Figure 5:**
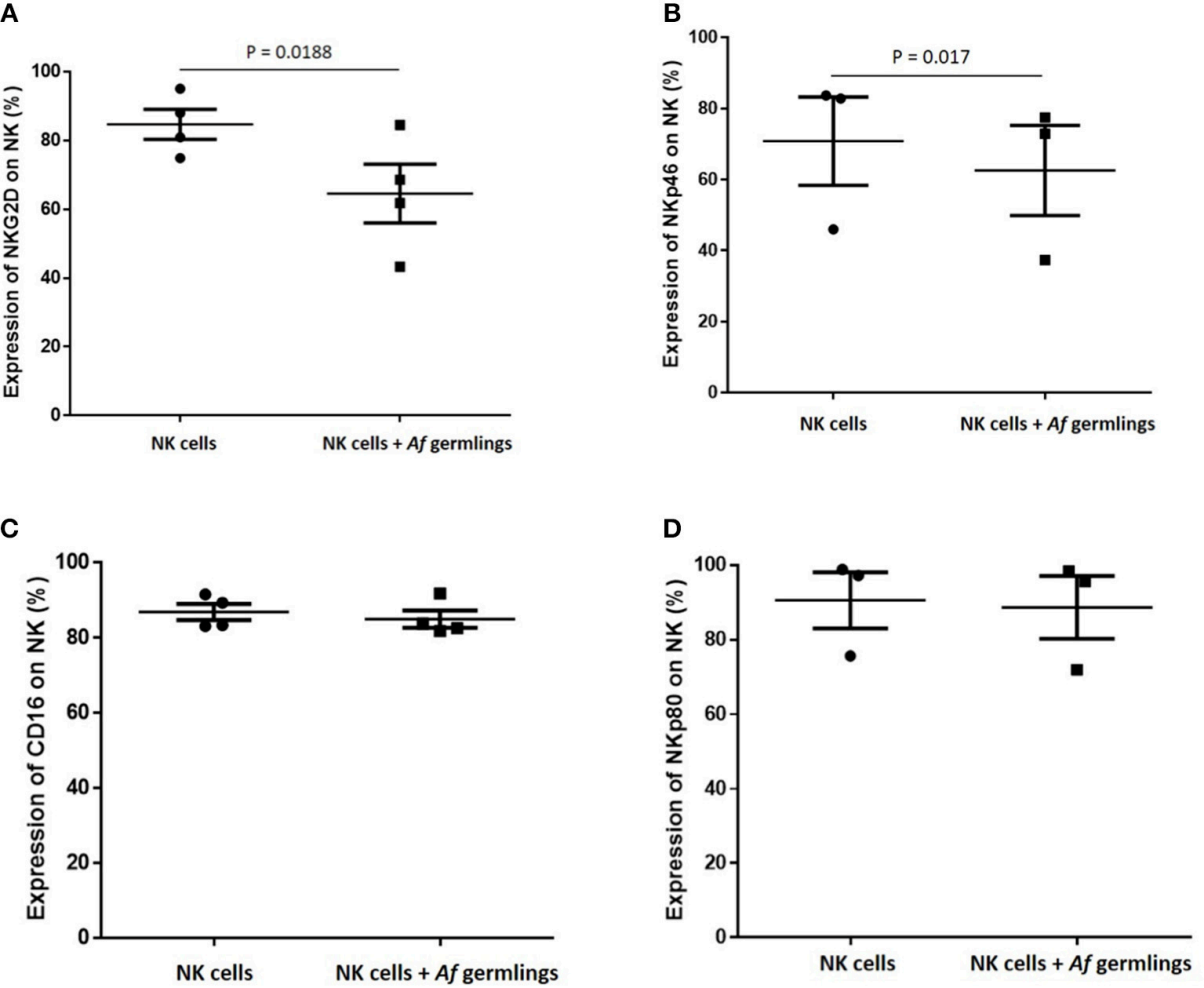
NK cell challenge with *A. fumigatus* reduces surface expression of the activatory receptors NKG2D and NKp46 **(A)**. NKG2D NK cell surface expression is reduced by *A. fumigatus*. Human NK cells were incubated overnight with *A. fumigatus* germlings at MOI = 0.05. NKG2D expression was determined by FACS, gating live NK cells. Statistical analysis was performed using the Student's paired *t*-test. *n* = 4 **(B)**. NKp46 NK cell surface expression is reduced by *A. fumigatus*. Human NK cells were incubated overnight with *A. fumigatus* germlings at MOI = 0.05. NKp46 expression was determined by FACS, gating live NK cells. Statistical analysis was performed using the Student's paired *t*-test. *n* = 3 **(C)**. CD16 expression on NK cells surface is not altered in the presence of *A. fumigatus*. Human NK cells were incubated overnight with *A. fumigatus* germlings at MOI = 0.05. CD16 expression was determined by FACS, gating live NK cells. Statistical analysis was performed using the Student's paired *t*-test. *n* = 4 **(D)**. NKp80 expression on NK cells surface is not altered in the presence of *A. fumigatus*. Human NK cells were incubated overnight with *A. fumigatus* germlings at MOI = 0.05. NKp80 expression was determined by FACS, gating live NK cells. Statistical analysis was performed using the Student's paired *t*-test. *n* = 3.

Next, we sought to characterise the influence of *A. fumigatus* interactions on NK cell activating receptors. Incubation of NK cells with *A. fumigatus in vitro* resulted in a significant reduction of expression of the activating receptors NKG2D (Figures 5A, S3) and NKp46 on NK cells (Figures 5B, S4). NKG2D NK cell surface expression was reduced on CD16^−^ and on CD16^+^ subsets in the presence of *A. fumigatus* (Figure S3).

These data suggest that these activating receptors are involved in the recognition of *A. fumigatus* by NK cells. No alterations in CD16, NKp80 expression (Figures 5C,D, S5), or NKp30, TLR2, and TLR4 (not shown) were observed. Further analysis revealed that NK cell challenge with *A. fumigatus* leads to increased cell surface CD107a expression on CD56dim CD16+ subset (Figure S6).

### NK cell interactions with *A. fumigatus* hyphae results in fungal DNA release

**Figure 6:**
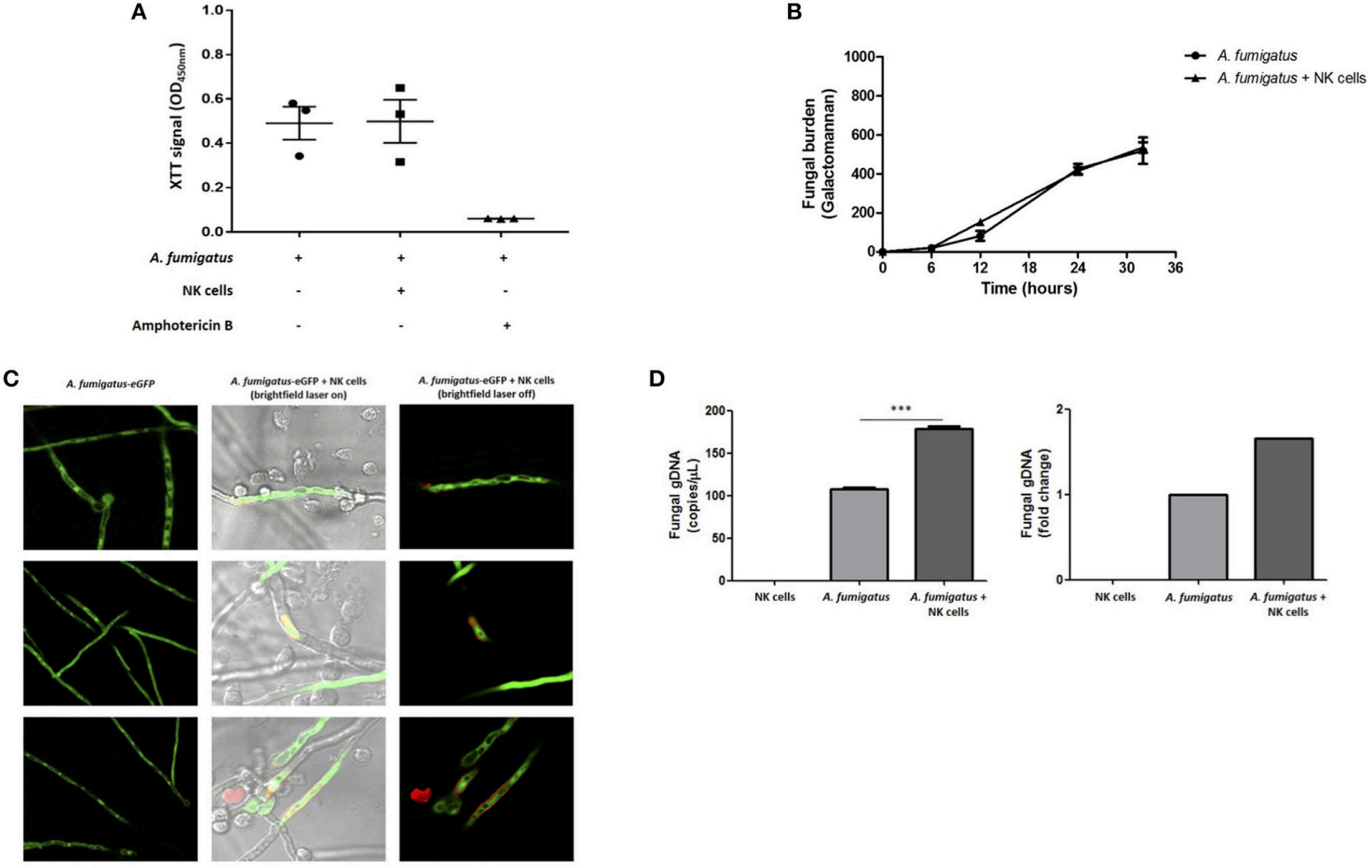
NK cell interaction with *A. fumigatus* hyphae results in fungal DNA release **(A)**. Analysis of fungal burden by XTT assay. *A. fumigatus* germlings were incubated overnight either in the absence or presence of NK cells at a MOI of 0.05. Graph shows the mean ± SEM of fungal burden. Statistical analysis was performed using the Student's paired *t*-test. *n* = 3 **(B)**. Analysis of fungal burden by galactomannan assay. *A. fumigatus* germlings were incubated overnight either in the absence or presence of NK cells at a MOI of 0.05. The culture supernatant was collected at different time points and galactomannan was determined. Graph shows the mean ± SEM of fungal burden. This data is representative of three independent experiments. Statistical analysis was performed using the Student's unpaired *t*-test. Y –axis is Galactomannan **(C)**. NK cell contact with *A. fumigatus* hyphae leads to DNA release. *A. fumigatus* germlings were incubated overnight in presence or absence of NK cells at MOI = 0.05. After the incubation period, propidium iodide was added to all wells and fungal viability was analysed by confocal microscopy. *A. fumigatus* are indicated in green and PI staining indicated in red. **(D)** NK cell interaction with *A. fumigatus* results in fungal DNA release. *A. fumigatus* germlings were incubated overnight in presence or absence of NK cells at MOI = 0.05. After the incubation period, culture supernatants were collected and fungal gDNA was quantified by qPCR. Results are shown in copies/μL and fold change. ***p < 0.001.

To systematically evaluate NK cell-mediated damage of *A. fumigatus*, we performed the XTT colorimetric assay, the PLATELIA *Aspergillus* galactomannan enzyme immunoassay and direct imaging using propidium iodide (PI) dye. The XTT assay did not show a significant difference in *A. fumigatus* metabolism when co-cultured with NK cells (Figure 6A). Additionally, co-incubation of *A. fumigatus* with NK cells did not inhibit fungal galactomannan production (14) (Figure 6B). However direct imaging during NK cell adhesion revealed DNA release at the *A. fumigatus* hyphal surface (Figure 6C) that was consistent between three donors. Concordantly there was a significant increase in fungal DNA release from *A. fumigatus* (Figure 6D), echoing previous studies of fungal damage during host defence or autolysis (15, 16).

### *Aspergillus fumigatus* impairs NK cell cytokine response to leukaemic cells

**Figure 7:**
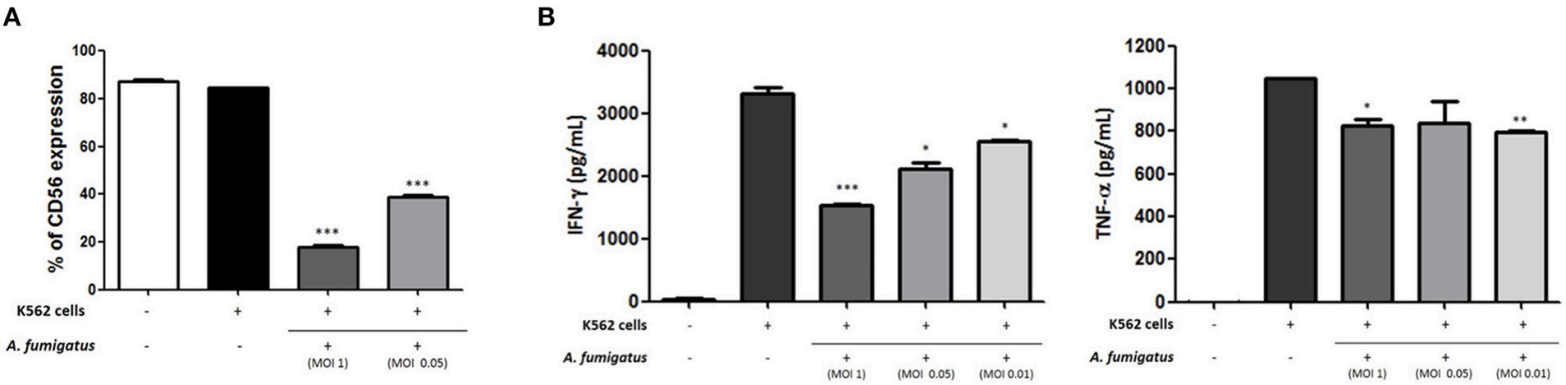
*Aspergillus fumigatus* impairs NK cell cytokine responses to leukaemic cells **(A)**. *A. fumigatus* reduces NK cell surface CD56 expression during co-incubation with the K562 leukaemic cell line. NK cells were incubated with K562 cell line at an effector:target ratio of 1:1 either in absence or presence of *A. fumigatus* resting conidia at different MOIs. Following overnight incubation, CD56 expression was determined by FACS, gating live NK cells. Bar graph shows the mean + SEM of CD56 expression. This data is representative of three independent experiments. Statistical analysis was performed using the Student's unpaired *t*-test. ***p < 0.001 **(B)**. *A. fumigatus* impairs NK cell cytokine response to K562 cell line. NK cells were incubated with K562 cell line at an effector:target ratio of 1:1 either in absence or presence of *A. fumigatus* resting conidia at different MOIs. Following overnight incubation, the supernatant of the co-cultures was collected and IFN-γ and TNF-α concentration was determined by ELISA. Bar graphs show the mean + SEM of IFN-γ and TNF-α production. These data are representative of three independent experiments. Statistical analysis was performed using the Student's unpaired *t*-test. *p < 0.05, **p < 0.01, ***p < 0.001.

As CD56 has an important role in target killing (12, 13) we hypothesised that *A. fumigatus* impaired NK cell responses to the leukaemia cell line K562. Co-incubation of *A. fumigatus* with NK cells and K562 leukaemic cells resulted in a significant down-regulation of CD56 expression (Figure 7A) and a significant reduction in cytokine production by NK cells in response to K562 (Figure 7B). For all donors, the presence of *A. fumigatus* induced a stronger inhibition of IFN-γ production in comparison with the inhibition observed for TNF-α release, which was MOI-dependent. Further studies revealed that the presence of *A. fumigatus* does not result in impaired NK cell cytotoxic response to K562 leukaemic cells (Figure S7).

## Discussion

As recent studies have further highlighted direct responses to NK cells for a range of fungi, we sought to systematically characterise NK cell responses to *A. fumigatus* in detail. Broadly onsistent with previous studies, we observed a weak NK cell IFN-γ and TNF-α response to *A. fumigatus*, (Figure 1) (8, 17). We also found that NK cells were able to respond to *A. fumigatus* through low-level chemokine responses via MIP-1α, MIP-1β, and RANTES. Defective cytokine production was associated with tight adherence to hyphae, associated with contact-dependent internalisation of the CD56 glycoprotein, confirming recent reports (18), but also importantly reduced cell surface expression of the known NK cell activatory receptors NKG2D and NKp46. NK cells exhibited weak direct fungal cytotoxicity characterised by granule polarisation at the hyphal synapse and fungal DNA release, but not fungal death. This combination of features is characteristic of an NK cell exhaustion phenotype (19). Finally, we showed that *A. fumigatus* severely impairs leukaemic responses of NK cells, suggesting that there could be clinically-relevant functional consequences for *A. fumigatus*-induced NK cell receptor modulation in clinically relevant populations. Conversely, AML patients did not display impaired degranulation responses to *A. fumigatus*.

The inhibitory effect of *A. fumigatus* on IFN-γ secretion by NK cells has been previously reported for IL-2 pre-stimulated NK cells (7). Interestingly, acquisition of CD56 and IFN-γ expression is synchronised during NK cell development (20). Moreover, CD56^bright^ CD16^−^ NK cell subsets have greater capacity to produce IFN-γ and TNF-α that is lost during NK cell maturation, and further studies have confirmed that CD56 spatially interacts with *A. fumigatus* (18, 21). Consistent with previous studies in neurons, we observed contact-dependent CD56 internalisation (22). Recent studies indicate that whilst translation of IFN-γ may occur in response to A. fumigatus hyphae, there is impaired release (17). The CD56 glycoprotein in NK cells is thought to be involved in both homophilic and heterophilic adhesion and target cell killing (12, 13). CD56^neg^ NK cell populations are expanded in chronic HIV-1 and HCV infection, and characterised by impaired capacity to kill target cells and to produce IFN-γ but maintaining the ability to produce chemokines (23, 24). This is consistent with NK cell exhaustion due to chronic antigen stimulation. Taken together this suggest that CD56 internalisation contributes to impaired NK cell responses to *A. fumigatus*.

NK cell contact with *A. fumigatus* also led to reduced cell surface expression of the major activating receptors NKG2D and NKp46. These observations suggest that there may be fungal ligands for these receptors, but that they fail to activate NK cells fully. Previous studies have also identified NKp46 as important for recognition of *Candida glabrata* (25). Furthermore, we found that *A. fumigatus* induced impaired NK cell cytokine response to the leukaemic cell line K562. This is particularly interesting, as it suggests that leukaemic patients with pulmonary aspergillosis may suffer the additional complication of impaired NK cell immune surveillance.

Confocal analysis of NK cell interactions with *A. fumigatus* showed that NK cells adhere to the fungus, accumulate F-actin at the immune synapse and polarise lytic granules, containing perforin and granulysin, toward *A. fumigatus*. Analysis of CD107a showed a significant increase in degranulation when NK cells were incubated with *A. fumigatus*. Our data suggests that degranulation occurs on the CD56^dim^ CD16^+^ subset (Figure S6). Our observations are in agreement with the literature that shows that the CD56^dim^ CD16^+^ subset represents the most differentiated and mature NK cells with high cytotoxicity capacity. However, there was no significant increase in perforin or granzyme B levels release in culture supernatants. This suggests that there may be a block in granule release at the NK-hyphal synapse. Polarisation of the lytic granules is not equivalent to a commitment to their secretion, with several more events being required before the granules fuse with the plasma membrane and their content is released into the synaptic cleft. Moreover, the fusion of granule content can be incomplete with the formation of a transient fusion pore at the plasma membrane accompanied by release of some but retention of most of the granule contents (26). It is possible that *A. fumigatus* may inhibit release of cytoxic granules by polarised NK cells. An alternative explanation is that perforin and granzyme B are released, but adhere strongly to fungal hyphae.

Incubation of NK cells with *A. fumigatus in vitro* induces the secretion of important chemokines, including MIP-1α, MIP-1β, and RANTES, that have been shown to play an important role as chemoattractants and coactivators for monocytes/macrophages and lymphocytes (27–29). These results suggest that NK cells may be central to the recruitment of other immune cells to the site of infection once they recognise the fungus in the host.

Consistent with this finding, the CD56^neg^ NK cell population observed in HIV and HCV patients maintain the ability to produce chemokines in response to targets (30). Regardless of the possible inhibitory effect of *A. fumigatus*, several studies have revealed a hierarchy in terms of the strength of the activating stimuli for induction of specific responses in NK cells. The analysis of phenotypic changes on NK cell surface showed that activating receptors such as NKG2D and NKp46 are down-regulated during synaptic adhesion of the the NK cell to *A. fumigatus*. However, this interaction does not seem to be strong enough to activate cytokine secretion and secretion of perforin or granzyme B, possibly as a consequence of receptor downregulation.

Analysis of NK cell antifungal activity showed that NK cells adhesion did not reduce fungal viability or inhibition of fungal metabolism; instead, NK cells induced fungal DNA release that is associated with fungal damage (15, 16). CpG-rich DNA motifs have been described as the natural ligands for TLR9 receptor (31–33) and deoxynucleic acids from *A. fumigatus, Candida albicans*, and *Cryptococcus neoformans* directly activate dendritic cells (34–36). We have also recently shown a key role for TLR9 in activating *A. fumigatus* macrophage response through a novel calcineurin- dependent pathway (37). Taken together these observations suggest that the interaction between NK cells and *A. fumigatus* leads to low-level hyphal damage and fungal DNA release. This is consistent with previous studies (7). This may be important for activation of subsequent systemic immune responses through nucleic acid-sensing pathways. NK cells may be a non-redundant second line of defence against invasive fungal infection in individuals with defects in host immunity.

In conclusion, human NK cells generate a partial response to *A. fumigatus in vitro*, characterised by formation of a NK cell-hyphal synapse, polarisation of lytic granules, chemokine release and low-level fungal damage. However, there is contact-dependent cell surface down-regulation of a number of NK cell activating receptors including NKG2D and NKp46 during NK cell interactions with *A. fumigatus*, associated with impaired TNF-α and IFN-ɤ responses and a failure of full granule release. These observations suggest that *A. fumigatus* mediates NK cell immunoparesis or exhaustion through a contact-dependent mechanism.

## Author contributions

DA-J, VS, PK, and KR designed the study. VS and TS conducted the experiments. All authors contributed to the writing of the manuscript.

### Conflict of interest statement

The authors declare that the research was conducted in the absence of any commercial or financial relationships that could be construed as a potential conflict of interest.
