# Supplementary material for: Human NK Cells Develop an Exhaustion Phenotype During Polar Degranulation at the Aspergillus fumigatus Hyphal Synapse
Source: Front Immunol. 2018 Oct 22;9:2344. doi: 10.3389/fimmu.2018.02344 (PMC6204393; doi:10.3389/fimmu.2018.02344)
Supplement: Supplementary file 1 [file Data_Sheet_1.docx]

**Supplementary Data**


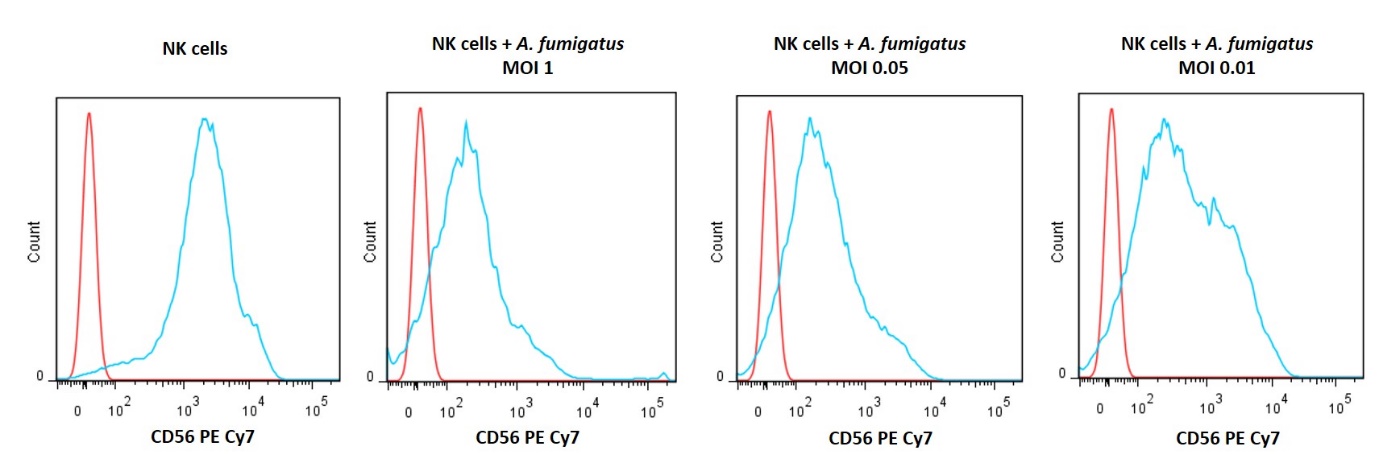


**Figure S1.** **Representative flow cytometry histograms of CD56 expression on NK cell surface.** Human NK cells were incubated overnight with *A. fumigatus* germlings at MOI=1, MOI=0.05 and MOI=0.01. The red lines on histograms represent unstained NK cells and blue lines on histograms represent NK cells at different incubation conditions, stained with CD56 PE Cy7 antibody. This data is representative of three independent experiments.


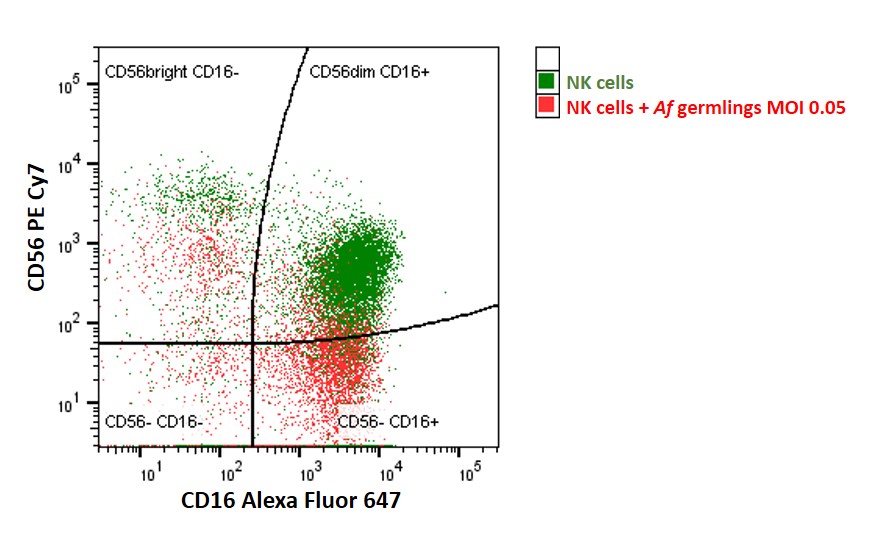

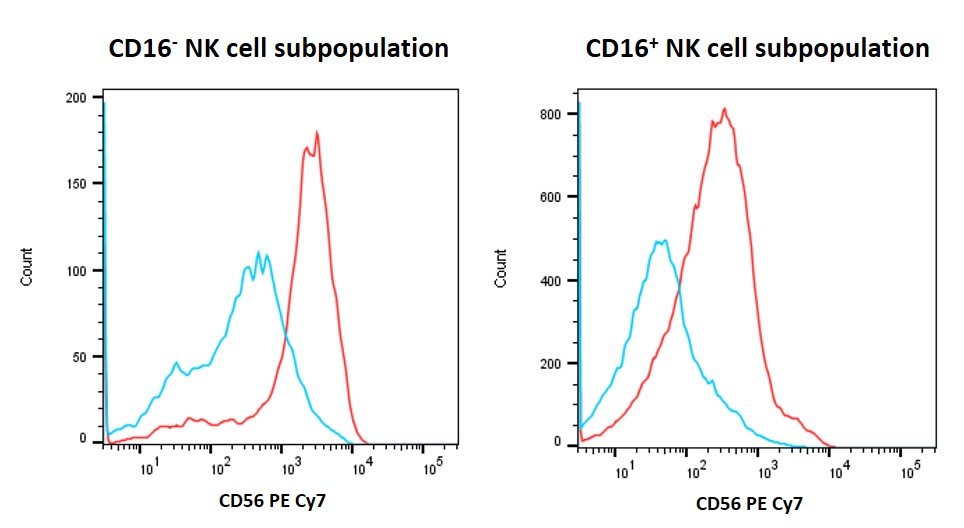


**Figure S2.** **CD56 NK cell surface expression is reduced on CD56^bright^ CD16^-^ and on CD56^dim^ CD16^+^ substes in the presence of *A. fumigatus*.** Human NK cells were incubated overnight with *A. fumigatus* germlings MOI=0.05. CD56 and CD16 expression was determined by FACS, gating live NK cells. The red lines on histograms represent CD56 mean fluorescence intensity on NK cells and blue lines on histograms represent CD56 mean fluorescence intensity on NK cells incubated with *A. fumigatus* germlings at MOI=0.05. This data is representative of three independent experiments.


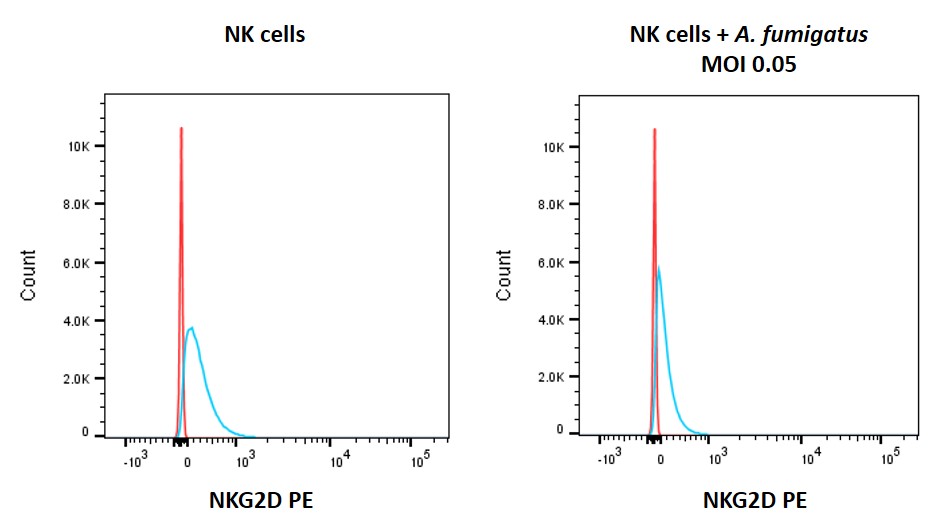


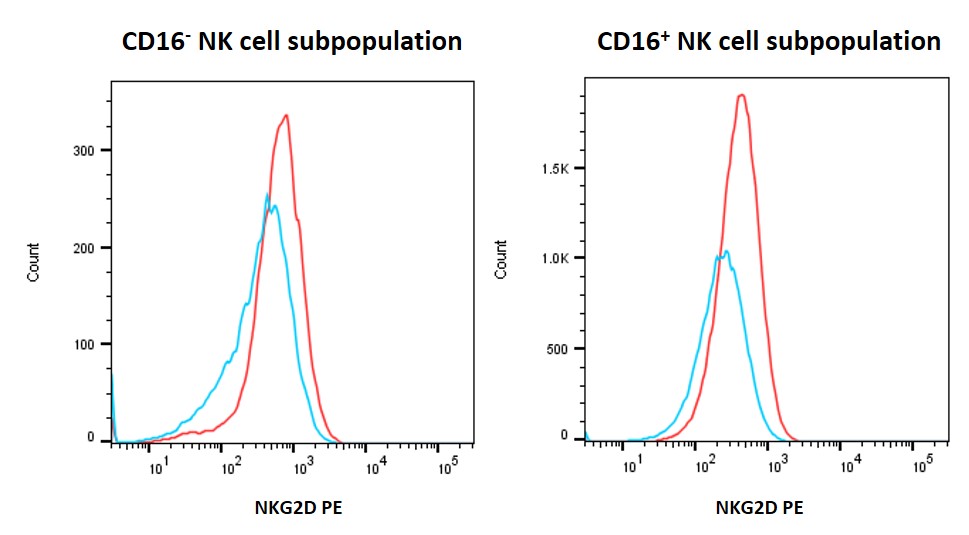


**Figure S3. NKG2D expression is reduced on NK cell surface in the presence of *A. fumigatus*.** Human NK cells were incubated overnight with *A. fumigatus* germlings at MOI=0.05. Top histograms show NKG2D expression on total NK cell population with the red lines representing unstained NK cells and blue lines representing NK cells at different incubation conditions. Bottom histograms show NKG2D expression on CD16^-^ and CD16^+^ NK cell subpopulations with the red lines representing NKG2D mean fluorescence intensity on NK cells and blue lines representing NKG2D mean fluorescence intensity on NK cells incubated with *A. fumigatus* germlings at MOI=0.05. This data is representative of four independent experiments.


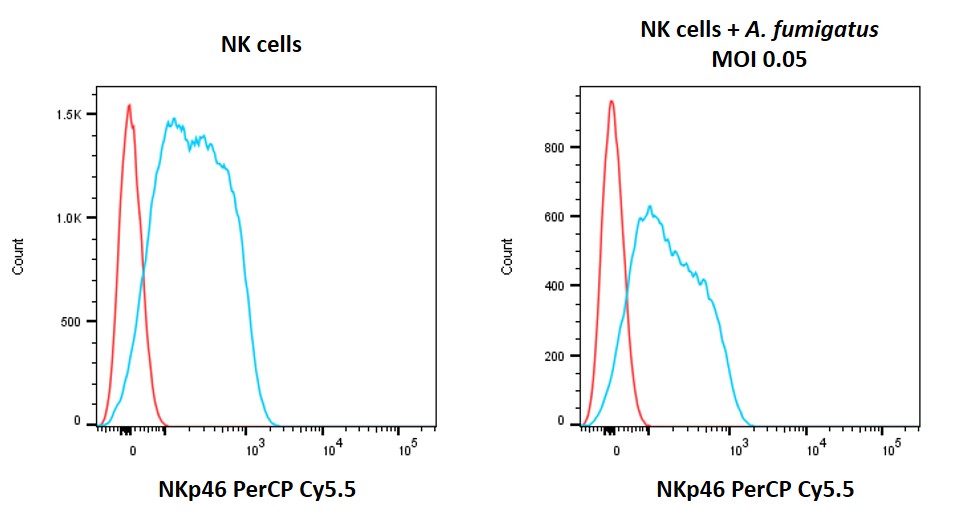


**Figure S4. Representative flow cytometry histograms of NKp46 expression on NK cell surface.** Human NK cells were incubated overnight with *A. fumigatus* germlings at MOI=0.05. The red lines on histograms represent unstained NK cells and blue lines on histograms represent NK cells at different incubation conditions, stained with NKp46 PerCP Cy5.5 antibody. This data is representative of three independent experiments.


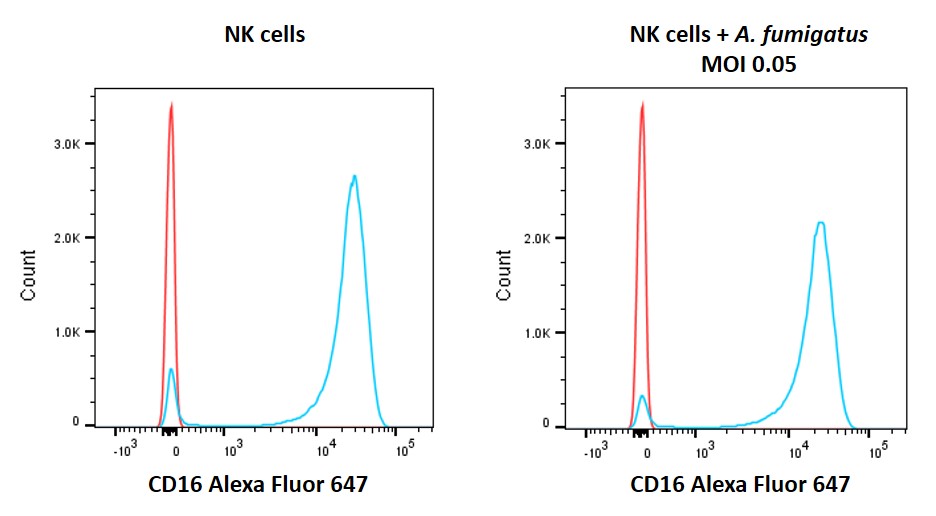


**Figure S5. Representative flow cytometry histograms of CD16 expression on NK cell surface.** Human NK cells were incubated overnight with *A. fumigatus* germlings at MOI=0.05. The red lines on histograms represent unstained NK cells and blue lines on histograms represent NK cells at different incubation conditions, stained with CD16 Alex Fluor 647 antibody. This data is representative of four independent experiments.


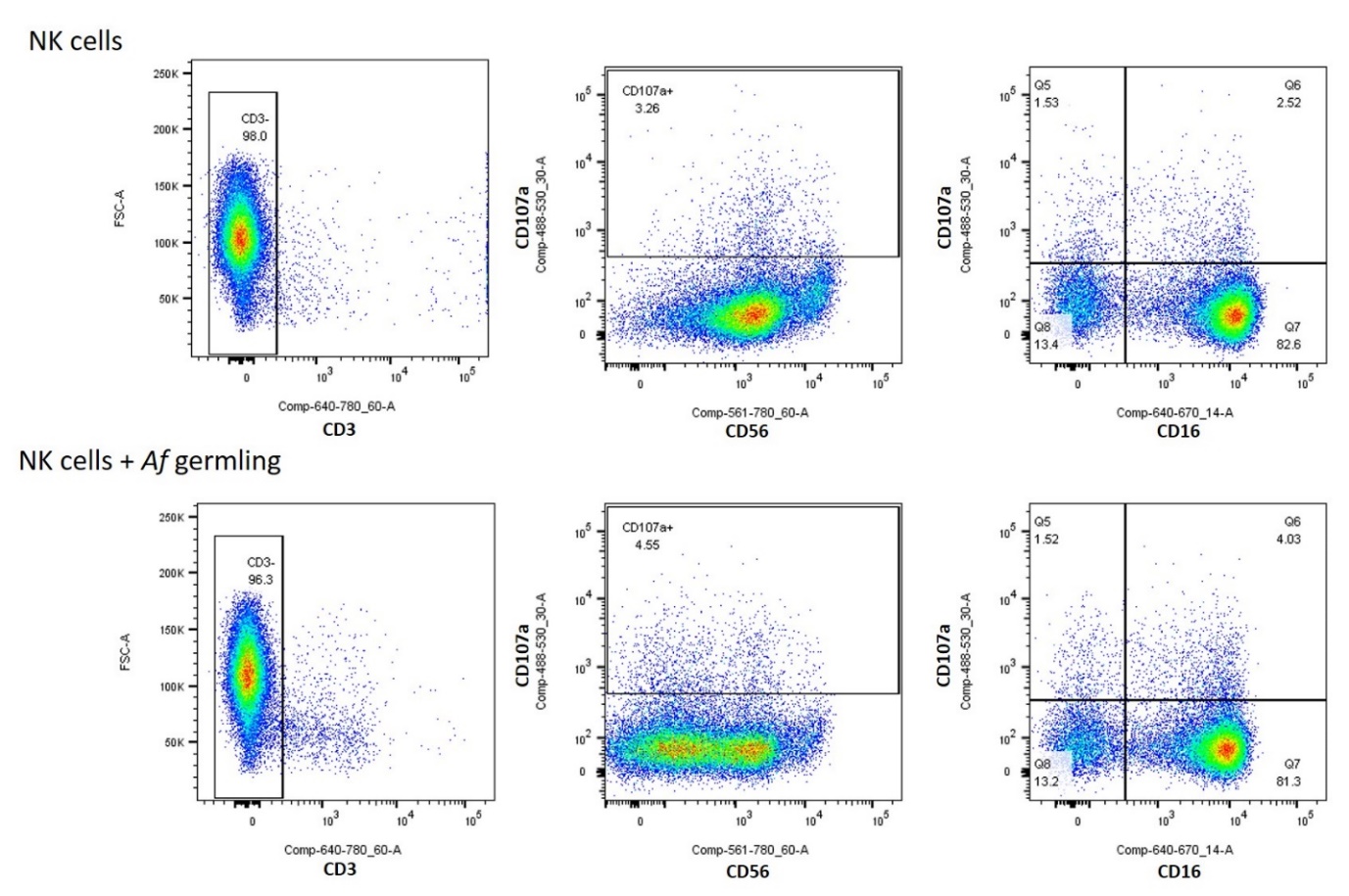


**Figure S6. NK cell challenge with *A. fumigatus* leads to increased cell surface CD107a expression on CD56dim CD16+ subset.** Isolated human NK cells were incubated overnight with *A. fumigatus* germlings at MOI=0.05. CD107a antibody was added at time 0 hours. After the incubation time, the cells were collected and washed with PBS. Cells were stained with CD3, CD56 and CD16 surface antibodies. CD107a expression by NK cells was determined by FACS analysis.


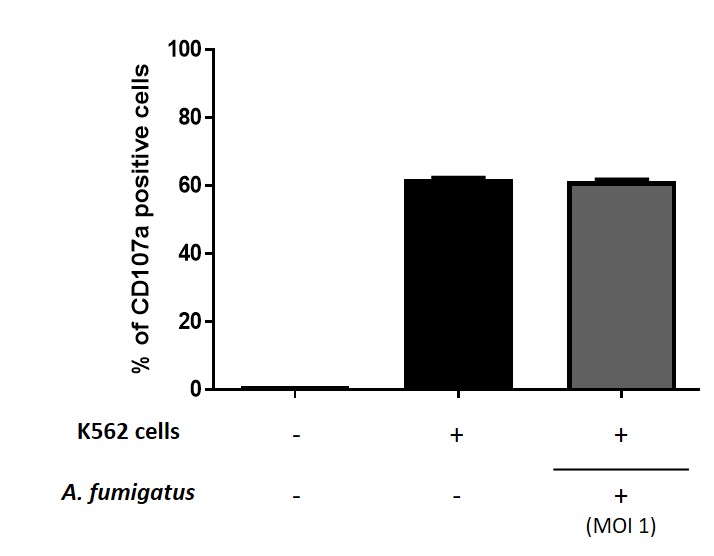


**Figure S7. *A. fumigatus* does not impair NK cell cytotoxic response to K562 cell line.** NK cells were incubated with K562 cell line at an effector:target ratio of 1:1 either in absence or presence of *A. fumigatus* resting conidia at MOI 1. CD107a antibody was added at time 0 hours. Following overnight incubation, the cells were collected and washed with PBS. Cells were stained with surface antibodies, followed by the staining with Aqua zombie dye. CD107a expression by NK cells was determined by FACS analysis. Graphs show the mean ± SEM of CD107a expression. Statistical analysis was performed using the Student’s paired t-test. This data is representative of three independent experiments.
